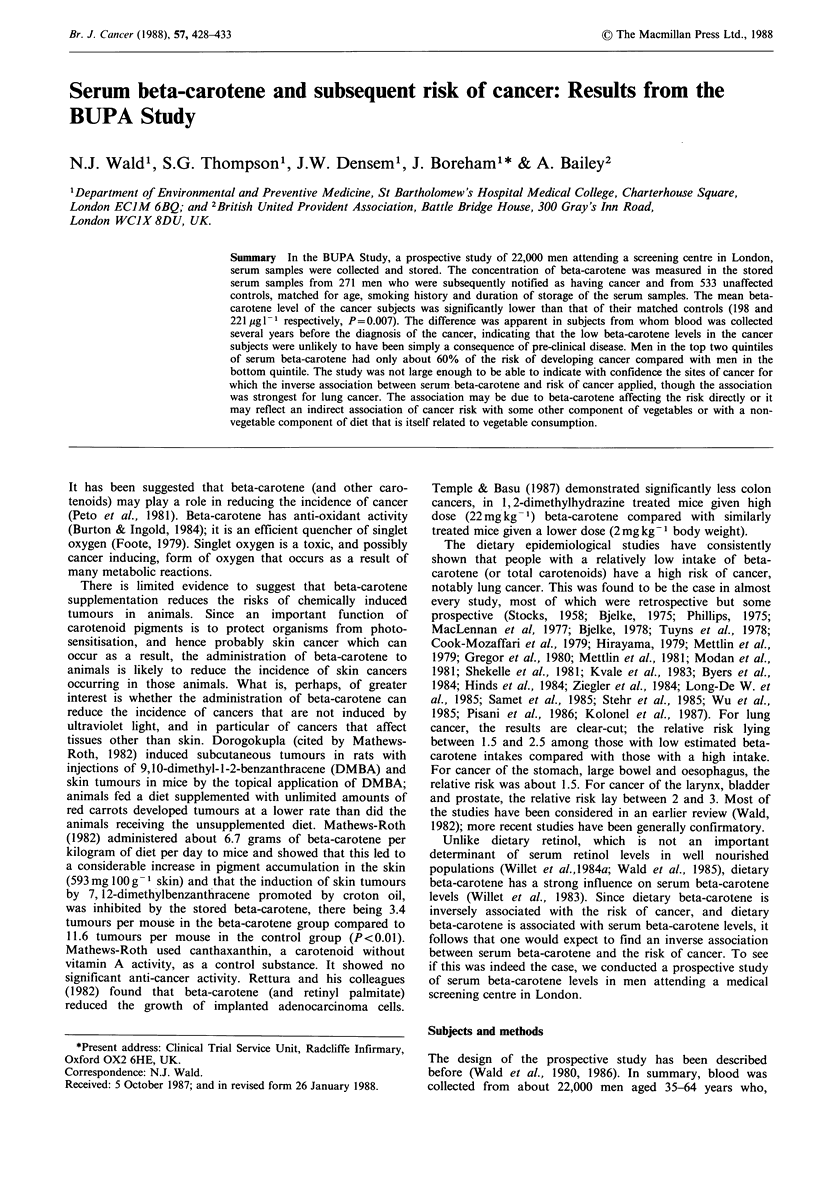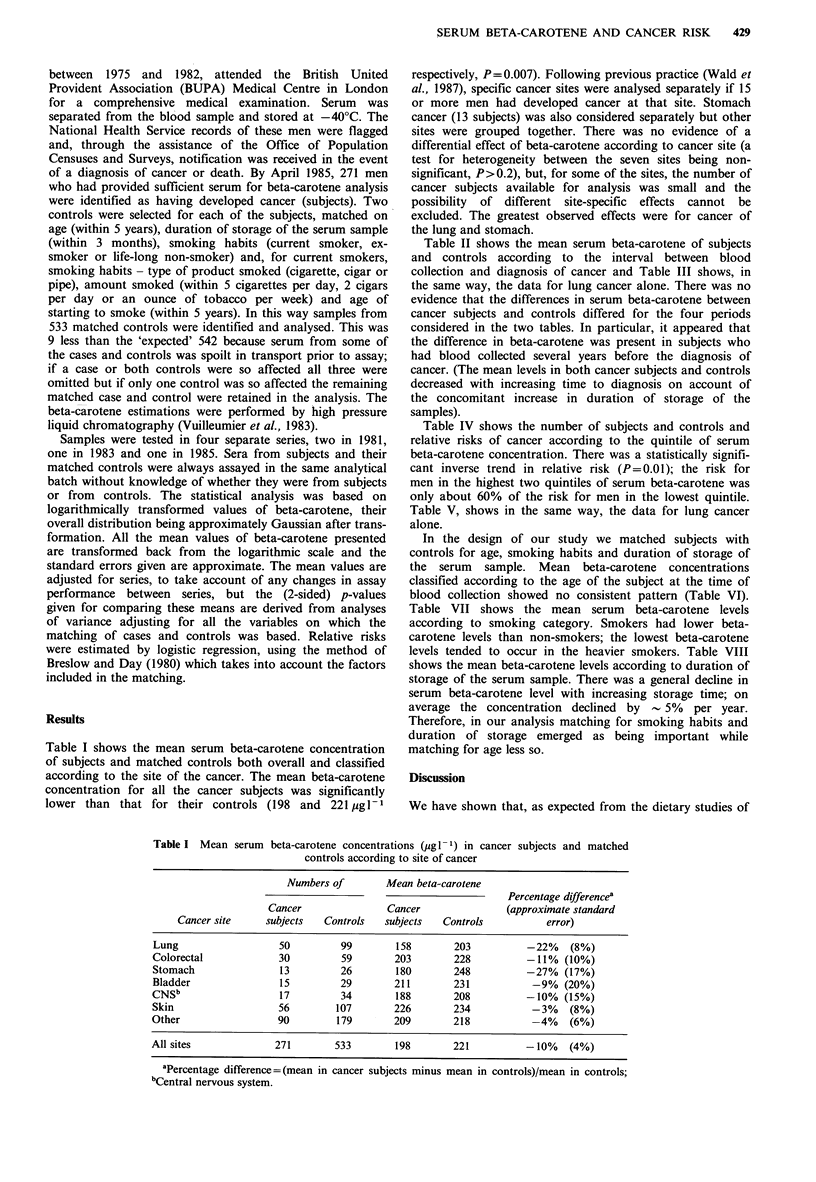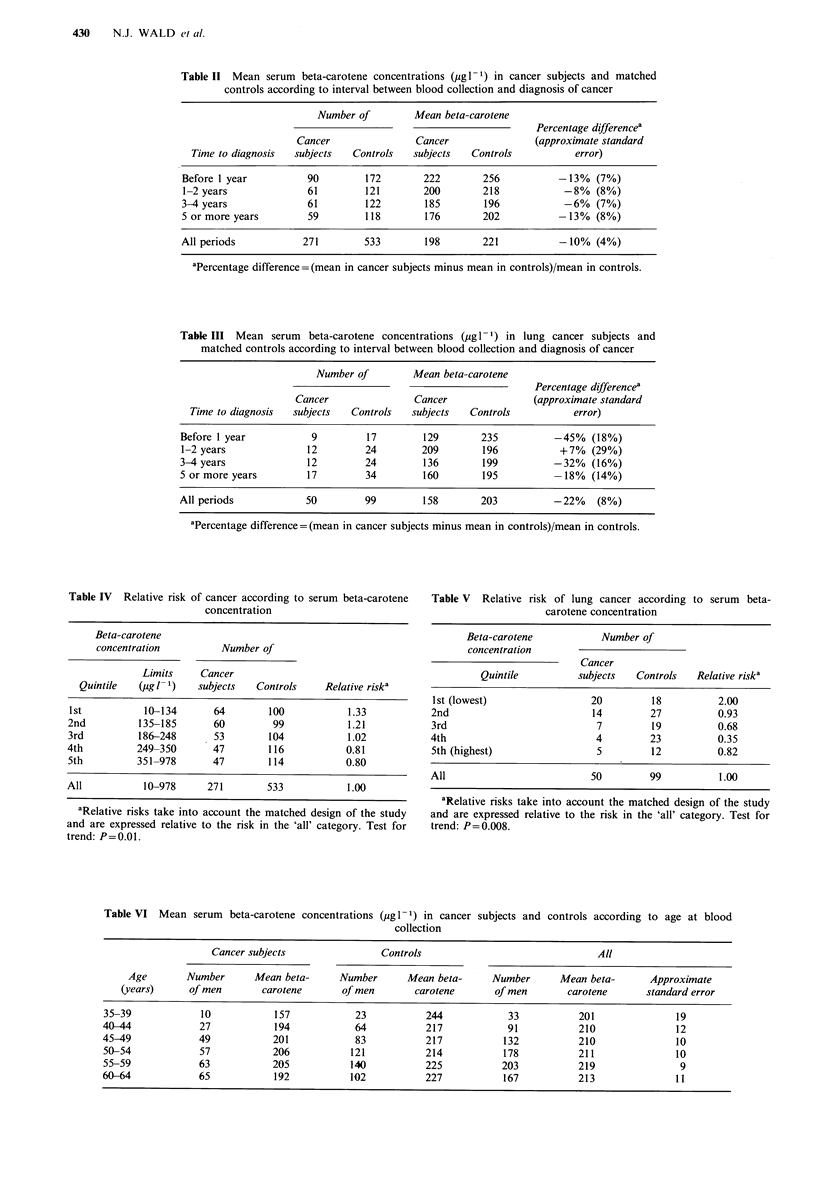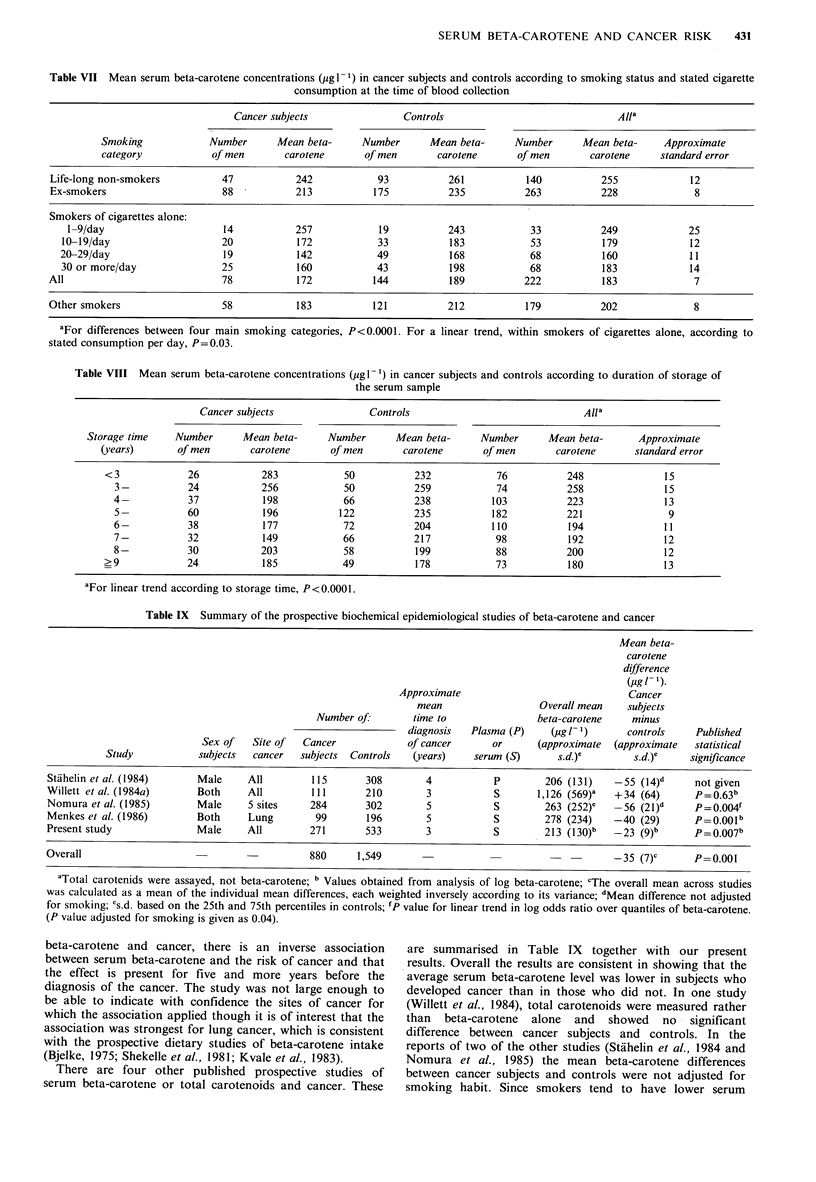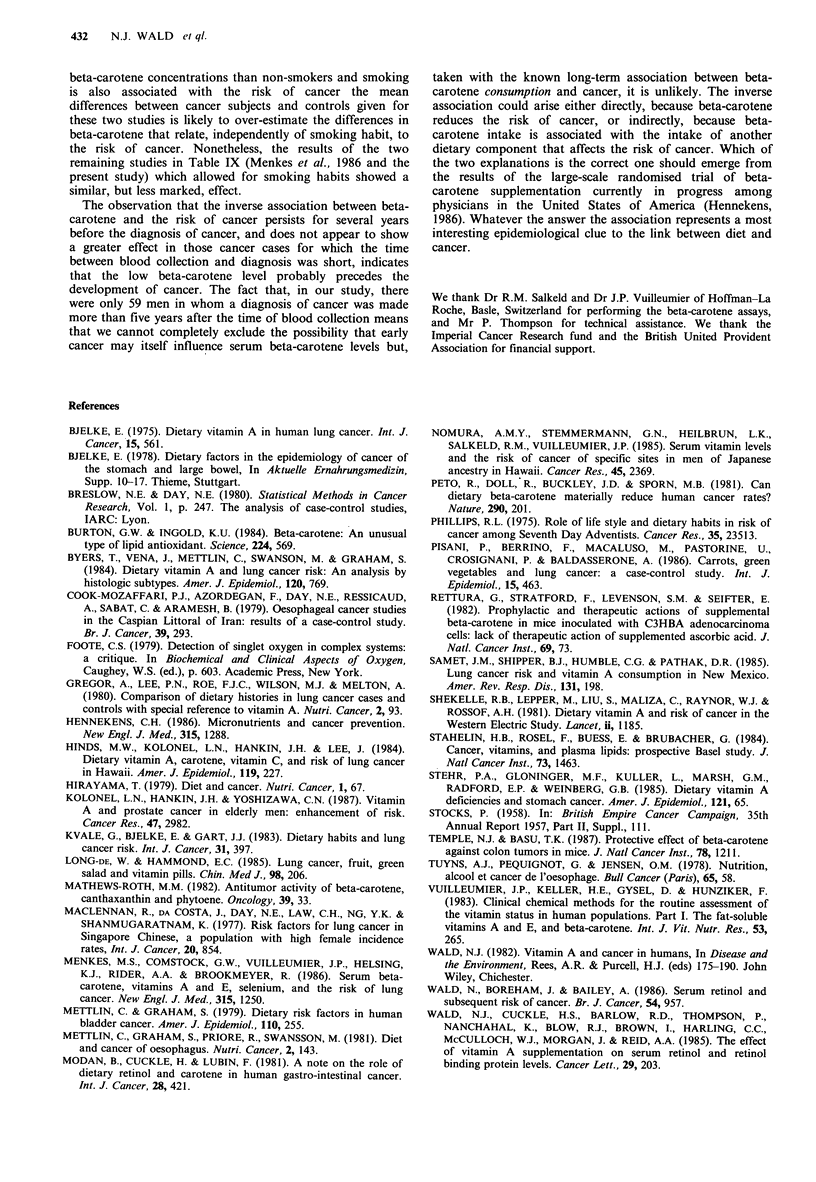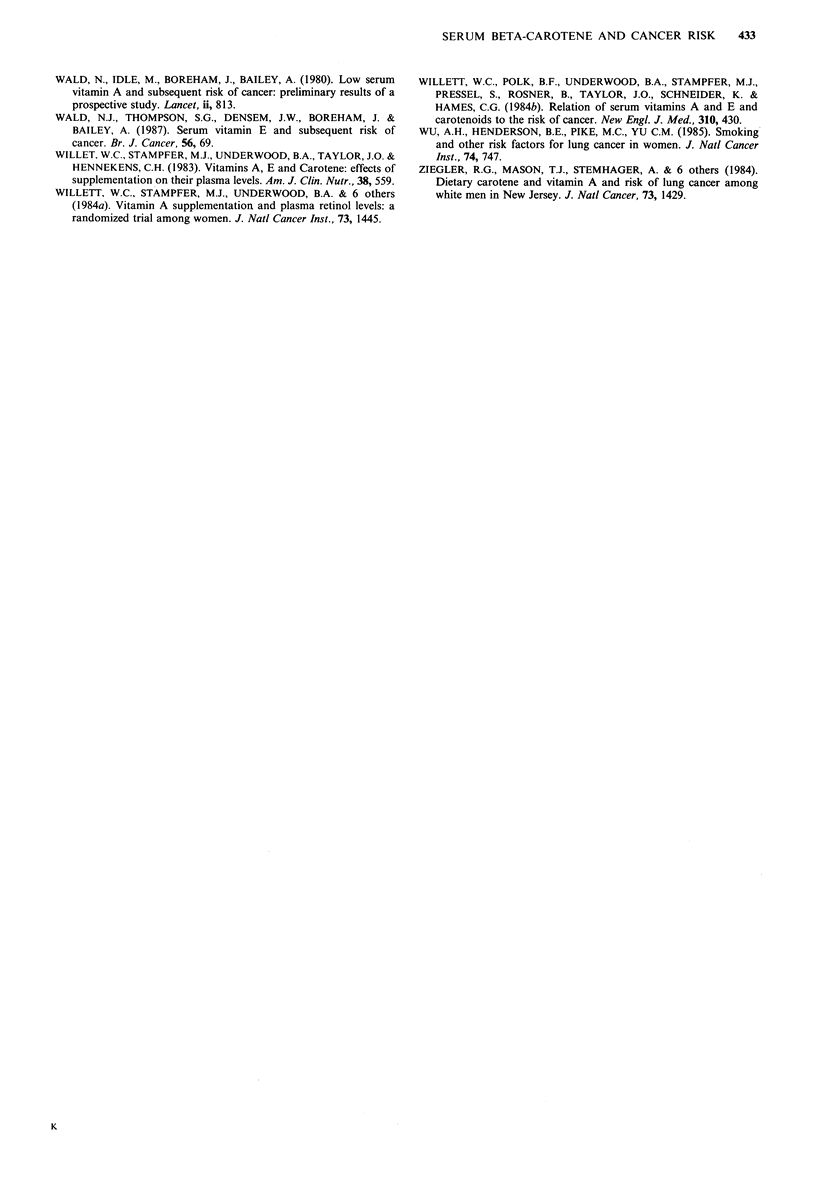# Serum beta-carotene and subsequent risk of cancer: results from the BUPA Study.

**DOI:** 10.1038/bjc.1988.97

**Published:** 1988-04

**Authors:** N. J. Wald, S. G. Thompson, J. W. Densem, J. Boreham, A. Bailey

**Affiliations:** Department of Environmental and Preventive Medicine, St Bartholomew's Hospital Medical College, London, UK.

## Abstract

In the BUPA Study, a prospective study of 22,000 men attending a screening centre in London, serum samples were collected and stored. The concentration of beta-carotene was measured in the stored serum samples from 271 men who were subsequently notified as having cancer and from 533 unaffected controls, matched for age, smoking history and duration of storage of the serum samples. The mean beta-carotene level of the cancer subjects was significantly lower than that of their matched controls (198 and 221 micrograms l-1 respectively, P = 0.007). The difference was apparent in subjects from whom blood was collected several years before the diagnosis of the cancer, indicating that the low beta-carotene levels in the cancer subjects were unlikely to have been simply a consequence of pre-clinical disease. Men in the top two quintiles of serum beta-carotene had only about 60% of the risk of developing cancer compared with men in the bottom quintile. The study was not large enough to be able to indicate with confidence the sites of cancer for which the inverse association between serum beta-carotene and risk of cancer applied, though the association was strongest for lung cancer. The association may be due to beta-carotene affecting the risk directly or it may reflect an indirect association of cancer risk with some other component of vegetables or with a nonvegetable component of diet that is itself related to vegetable consumption.


Br. J. Cancer (1988), 57, 428–433 © The Macmillan Press Ltd., 1988

### Serum beta-carotene and subsequent risk of cancer: Results from the BUPA Study

N.J. Wald[1], S.G. Thompson[1], J.W. Densem[1], J. Boreham[1]* & A. Bailey[2]

[1]*Department of Environmental and Preventive Medicine, St Bartholomew's Hospital Medical College, Charterhouse Square, London EC1M 6BQ; and* [2]*British United Provident Association, Battle Bridge House, 300 Gray's Inn Road, London WC1X 8DU, UK.*

**Summary** In the BUPA Study, a prospective study of 22,000 men attending a screening centre in London, serum samples were collected and stored. The concentration of beta-carotene was measured in the stored serum samples from 271 men who were subsequently notified as having cancer and from 533 unaffected controls, matched for age, smoking history and duration of storage of the serum samples. The mean beta-carotene level of the cancer subjects was significantly lower than that of their matched controls (198 and 221 $\mu g l^{-1}$ respectively, $P=0.007$). The difference was apparent in subjects from whom blood was collected several years before the diagnosis of the cancer, indicating that the low beta-carotene levels in the cancer subjects were unlikely to have been simply a consequence of pre-clinical disease. Men in the top two quintiles of serum beta-carotene had only about 60% of the risk of developing cancer compared with men in the bottom quintile. The study was not large enough to be able to indicate with confidence the sites of cancer for which the inverse association between serum beta-carotene and risk of cancer applied, though the association was strongest for lung cancer. The association may be due to beta-carotene affecting the risk directly or it may reflect an indirect association of cancer risk with some other component of vegetables or with a non-vegetable component of diet that is itself related to vegetable consumption.

It has been suggested that beta-carotene (and other carotenoids) may play a role in reducing the incidence of cancer (Peto *et al.,* 1981). Beta-carotene has anti-oxidant activity (Burton & Ingold, 1984); it is an efficient quencher of singlet oxygen (Foote, 1979). Singlet oxygen is a toxic, and possibly cancer inducing, form of oxygen that occurs as a result of many metabolic reactions.

There is limited evidence to suggest that beta-carotene supplementation reduces the risks of chemically induced tumours in animals. Since an important function of carotenoid pigments is to protect organisms from photosensitisation, and hence probably skin cancer which can occur as a result, the administration of beta-carotene to animals is likely to reduce the incidence of skin cancers occurring in those animals. What is, perhaps, of greater interest is whether the administration of beta-carotene can reduce the incidence of cancers that are not induced by ultraviolet light, and in particular of cancers that affect tissues other than skin. Dorogokupla (cited by Mathews-Roth, 1982) induced subcutaneous tumours in rats with injections of 9,10-dimethyl-1-2-benzanthracene (DMBA) and skin tumours in mice by the topical application of DMBA; animals fed a diet supplemented with unlimited amounts of red carrots developed tumours at a lower rate than did the animals receiving the unsupplemented diet. Mathews-Roth (1982) administered about 6.7 grams of beta-carotene per kilogram of diet per day to mice and showed that this led to a considerable increase in pigment accumulation in the skin (593 mg 100 $g^{-1}$ skin) and that the induction of skin tumours by 7, 12-dimethylbenzanthracene promoted by croton oil, was inhibited by the stored beta-carotene, there being 3.4 tumours per mouse in the beta-carotene group compared to 11.6 tumours per mouse in the control group ($P<0.01$). Mathews-Roth used canthaxanthin, a carotenoid without vitamin A activity, as a control substance. It showed no significant anti-cancer activity. Rettura and his colleagues (1982) found that beta-carotene (and retinyl palmitate) reduced the growth of implanted adenocarcinoma cells. Temple & Basu (1987) demonstrated significantly less colon cancers, in 1, 2-dimethylhydrazine treated mice given high dose (22 mg $kg^{-1}$) beta-carotene compared with similarly treated mice given a lower dose (2 mg $kg^{-1}$ body weight).

The dietary epidemiological studies have consistently shown that people with a relatively low intake of beta-carotene (or total carotenoids) have a high risk of cancer, notably lung cancer. This was found to be the case in almost every study, most of which were retrospective but some prospective (Stocks, 1958; Bjelke, 1975; Phillips, 1975; MacLennan *et al,* 1977; Bjelke, 1978; Tuyns *et al.,* 1978; Cook-Mozaffari *et al.,* 1979; Hirayama, 1979; Mettlin *et al.,* 1979; Gregor *et al.,* 1980; Mettlin *et al.,* 1981; Modan *et al.,* 1981; Shekelle *et al.,* 1981; Kvale *et al.,* 1983; Byers *et al.,* 1984; Hinds *et al.,* 1984; Ziegler *et al.,* 1984; Long-De W. *et al.,* 1985; Samet *et al.,* 1985; Stehr *et al.,* 1985; Wu *et al.,* 1985; Pisani *et al.,* 1986; Kolonel *et al.,* 1987). For lung cancer, the results are clear-cut; the relative risk lying between 1.5 and 2.5 among those with low estimated beta-carotene intakes compared with those with a high intake. For cancer of the stomach, large bowel and oesophagus, the relative risk was about 1.5. For cancer of the larynx, bladder and prostate, the relative risk lay between 2 and 3. Most of the studies have been considered in an earlier review (Wald, 1982); more recent studies have been generally confirmatory.

Unlike dietary retinol, which is not an important determinant of serum retinol levels in well nourished populations (Willet *et al.,*1984*a*; Wald *et al.,* 1985), dietary beta-carotene has a strong influence on serum beta-carotene levels (Willet *et al.,* 1983). Since dietary beta-carotene is inversely associated with the risk of cancer, and dietary beta-carotene is associated with serum beta-carotene levels, it follows that one would expect to find an inverse association between serum beta-carotene and the risk of cancer. To see if this was indeed the case, we conducted a prospective study of serum beta-carotene levels in men attending a medical screening centre in London.

### Subjects and methods

The design of the prospective study has been described before (Wald *et al.,* 1980, 1986). In summary, blood was collected from about 22,000 men aged 35–64 years who,

*Present address: Clinical Trial Service Unit, Radcliffe Infirmary, Oxford OX2 6HE, UK.
Correspondence: N.J. Wald.
Received: 5 October 1987; and in revised form 26 January 1988.

between 1975 and 1982, attended the British United Provident Association (BUPA) Medical Centre in London for a comprehensive medical examination. Serum was separated from the blood sample and stored at −40°C. The National Health Service records of these men were flagged and, through the assistance of the Office of Population Censuses and Surveys, notification was received in the event of a diagnosis of cancer or death. By April 1985, 271 men who had provided sufficient serum for beta-carotene analysis were identified as having developed cancer (subjects). Two controls were selected for each of the subjects, matched on age (within 5 years), duration of storage of the serum sample (within 3 months), smoking habits (current smoker, ex-smoker or life-long non-smoker) and, for current smokers, smoking habits – type of product smoked (cigarette, cigar or pipe), amount smoked (within 5 cigarettes per day, 2 cigars per day or an ounce of tobacco per week) and age of starting to smoke (within 5 years). In this way samples from 533 matched controls were identified and analysed. This was 9 less than the 'expected' 542 because serum from some of the cases and controls was spoilt in transport prior to assay; if a case or both controls were so affected all three were omitted but if only one control was so affected the remaining matched case and control were retained in the analysis. The beta-carotene estimations were performed by high pressure liquid chromatography (Vuilleumier *et al.*, 1983).

Samples were tested in four separate series, two in 1981, one in 1983 and one in 1985. Sera from subjects and their matched controls were always assayed in the same analytical batch without knowledge of whether they were from subjects or from controls. The statistical analysis was based on logarithmically transformed values of beta-carotene, their overall distribution being approximately Gaussian after transformation. All the mean values of beta-carotene presented are transformed back from the logarithmic scale and the standard errors given are approximate. The mean values are adjusted for series, to take account of any changes in assay performance between series, but the (2-sided) $p$-values given for comparing these means are derived from analyses of variance adjusting for all the variables on which the matching of cases and controls was based. Relative risks were estimated by logistic regression, using the method of Breslow and Day (1980) which takes into account the factors included in the matching.

### Results

Table I shows the mean serum beta-carotene concentration of subjects and matched controls both overall and classified according to the site of the cancer. The mean beta-carotene concentration for all the cancer subjects was significantly lower than that for their controls (198 and 221 $\mu g l^{-1}$ respectively, $P = 0.007$). Following previous practice (Wald *et al.*, 1987), specific cancer sites were analysed separately if 15 or more men had developed cancer at that site. Stomach cancer (13 subjects) was also considered separately but other sites were grouped together. There was no evidence of a differential effect of beta-carotene according to cancer site (a test for heterogeneity between the seven sites being non-significant, $P > 0.2$), but, for some of the sites, the number of cancer subjects available for analysis was small and the possibility of different site-specific effects cannot be excluded. The greatest observed effects were for cancer of the lung and stomach.

Table II shows the mean serum beta-carotene of subjects and controls according to the interval between blood collection and diagnosis of cancer and Table III shows, in the same way, the data for lung cancer alone. There was no evidence that the differences in serum beta-carotene between cancer subjects and controls differed for the four periods considered in the two tables. In particular, it appeared that the difference in beta-carotene was present in subjects who had blood collected several years before the diagnosis of cancer. (The mean levels in both cancer subjects and controls decreased with increasing time to diagnosis on account of the concomitant increase in duration of storage of the samples).

Table IV shows the number of subjects and controls and relative risks of cancer according to the quintile of serum beta-carotene concentration. There was a statistically significant inverse trend in relative risk ($P = 0.01$); the risk for men in the highest two quintiles of serum beta-carotene was only about 60% of the risk for men in the lowest quintile. Table V, shows in the same way, the data for lung cancer alone.

In the design of our study we matched subjects with controls for age, smoking habits and duration of storage of the serum sample. Mean beta-carotene concentrations classified according to the age of the subject at the time of blood collection showed no consistent pattern (Table VI). Table VII shows the mean serum beta-carotene levels according to smoking category. Smokers had lower beta-carotene levels than non-smokers; the lowest beta-carotene levels tended to occur in the heavier smokers. Table VIII shows the mean beta-carotene levels according to duration of storage of the serum sample. There was a general decline in serum beta-carotene level with increasing storage time; on average the concentration declined by $\sim 5\%$ per year. Therefore, in our analysis matching for smoking habits and duration of storage emerged as being important while matching for age less so.

### Discussion

We have shown that, as expected from the dietary studies of

**Table I** Mean serum beta-carotene concentrations ($\mu g l^{-1}$) in cancer subjects and matched controls according to site of cancer

| Cancer site | *Numbers of* Cancer subjects | Controls | *Mean beta-carotene* Cancer subjects | Controls | *Percentage difference*[a] *(approximate standard error)* |
|---|---|---|---|---|---|
| Lung | 50 | 99 | 158 | 203 | −22% (8%) |
| Colorectal | 30 | 59 | 203 | 228 | −11% (10%) |
| Stomach | 13 | 26 | 180 | 248 | −27% (17%) |
| Bladder | 15 | 29 | 211 | 231 | −9% (20%) |
| CNS[b] | 17 | 34 | 188 | 208 | −10% (15%) |
| Skin | 56 | 107 | 226 | 234 | −3% (8%) |
| Other | 90 | 179 | 209 | 218 | −4% (6%) |
| All sites | 271 | 533 | 198 | 221 | −10% (4%) |

[a]Percentage difference = (mean in cancer subjects minus mean in controls)/mean in controls; [b]Central nervous system.

**Table II** Mean serum beta-carotene concentrations ($\mu g l^{-1}$) in cancer subjects and matched controls according to interval between blood collection and diagnosis of cancer

| | Number of | | Mean beta-carotene | | |
|---|---|---|---|---|---|
| *Time to diagnosis* | *Cancer subjects* | *Controls* | *Cancer subjects* | *Controls* | *Percentage difference*[a] *(approximate standard error)* |
| Before 1 year | 90 | 172 | 222 | 256 | −13% (7%) |
| 1–2 years | 61 | 121 | 200 | 218 | −8% (8%) |
| 3–4 years | 61 | 122 | 185 | 196 | −6% (7%) |
| 5 or more years | 59 | 118 | 176 | 202 | −13% (8%) |
| All periods | 271 | 533 | 198 | 221 | −10% (4%) |

[a]Percentage difference = (mean in cancer subjects minus mean in controls)/mean in controls.

**Table III** Mean serum beta-carotene concentrations ($\mu g l^{-1}$) in lung cancer subjects and matched controls according to interval between blood collection and diagnosis of cancer

| | Number of | | Mean beta-carotene | | |
|---|---|---|---|---|---|
| *Time to diagnosis* | *Cancer subjects* | *Controls* | *Cancer subjects* | *Controls* | *Percentage difference*[a] *(approximate standard error)* |
| Before 1 year | 9 | 17 | 129 | 235 | −45% (18%) |
| 1–2 years | 12 | 24 | 209 | 196 | +7% (29%) |
| 3–4 years | 12 | 24 | 136 | 199 | −32% (16%) |
| 5 or more years | 17 | 34 | 160 | 195 | −18% (14%) |
| All periods | 50 | 99 | 158 | 203 | −22% (8%) |

[a]Percentage difference = (mean in cancer subjects minus mean in controls)/mean in controls.

**Table IV** Relative risk of cancer according to serum beta-carotene concentration

| Beta-carotene concentration | | Number of | | |
|---|---|---|---|---|
| *Quintile* | *Limits* *($\mu g l^{-1}$)* | *Cancer subjects* | *Controls* | *Relative risk*[a] |
| 1st | 10–134 | 64 | 100 | 1.33 |
| 2nd | 135–185 | 60 | 99 | 1.21 |
| 3rd | 186–248 | 53 | 104 | 1.02 |
| 4th | 249–350 | 47 | 116 | 0.81 |
| 5th | 351–978 | 47 | 114 | 0.80 |
| All | 10–978 | 271 | 533 | 1.00 |

[a]Relative risks take into account the matched design of the study and are expressed relative to the risk in the 'all' category. Test for trend: $P = 0.01$.

**Table V** Relative risk of lung cancer according to serum beta-carotene concentration

| Beta-carotene concentration | Number of | | |
|---|---|---|---|
| *Quintile* | *Cancer subjects* | *Controls* | *Relative risk*[a] |
| 1st (lowest) | 20 | 18 | 2.00 |
| 2nd | 14 | 27 | 0.93 |
| 3rd | 7 | 19 | 0.68 |
| 4th | 4 | 23 | 0.35 |
| 5th (highest) | 5 | 12 | 0.82 |
| All | 50 | 99 | 1.00 |

[a]Relative risks take into account the matched design of the study and are expressed relative to the risk in the 'all' category. Test for trend: $P = 0.008$.

**Table VI** Mean serum beta-carotene concentrations ($\mu g l^{-1}$) in cancer subjects and controls according to age at blood collection

| | Cancer subjects | | Controls | | All | | |
|---|---|---|---|---|---|---|---|
| *Age (years)* | *Number of men* | *Mean beta-carotene* | *Number of men* | *Mean beta-carotene* | *Number of men* | *Mean beta-carotene* | *Approximate standard error* |
| 35–39 | 10 | 157 | 23 | 244 | 33 | 201 | 19 |
| 40–44 | 27 | 194 | 64 | 217 | 91 | 210 | 12 |
| 45–49 | 49 | 201 | 83 | 217 | 132 | 210 | 10 |
| 50–54 | 57 | 206 | 121 | 214 | 178 | 211 | 10 |
| 55–59 | 63 | 205 | 140 | 225 | 203 | 219 | 9 |
| 60–64 | 65 | 192 | 102 | 227 | 167 | 213 | 11 |

**Table VII** Mean serum beta-carotene concentrations ($\mu g l^{-1}$) in cancer subjects and controls according to smoking status and stated cigarette consumption at the time of blood collection

| | *Cancer subjects* | | *Controls* | | *All*[a] | | |
|---|---|---|---|---|---|---|---|
| *Smoking category* | *Number of men* | *Mean beta-carotene* | *Number of men* | *Mean beta-carotene* | *Number of men* | *Mean beta-carotene* | *Approximate standard error* |
| Life-long non-smokers | 47 | 242 | 93 | 261 | 140 | 255 | 12 |
| Ex-smokers | 88 | 213 | 175 | 235 | 263 | 228 | 8 |
| Smokers of cigarettes alone: | | | | | | | |
| 1–9/day | 14 | 257 | 19 | 243 | 33 | 249 | 25 |
| 10–19/day | 20 | 172 | 33 | 183 | 53 | 179 | 12 |
| 20–29/day | 19 | 142 | 49 | 168 | 68 | 160 | 11 |
| 30 or more/day | 25 | 160 | 43 | 198 | 68 | 183 | 14 |
| All | 78 | 172 | 144 | 189 | 222 | 183 | 7 |
| Other smokers | 58 | 183 | 121 | 212 | 179 | 202 | 8 |

[a]For differences between four main smoking categories, $P<0.0001$. For a linear trend, within smokers of cigarettes alone, according to stated consumption per day, $P=0.03$.

**Table VIII** Mean serum beta-carotene concentrations ($\mu g l^{-1}$) in cancer subjects and controls according to duration of storage of the serum sample

| | *Cancer subjects* | | *Controls* | | *All*[a] | | |
|---|---|---|---|---|---|---|---|
| *Storage time (years)* | *Number of men* | *Mean beta-carotene* | *Number of men* | *Mean beta-carotene* | *Number of men* | *Mean beta-carotene* | *Approximate standard error* |
| <3 | 26 | 283 | 50 | 232 | 76 | 248 | 15 |
| 3– | 24 | 256 | 50 | 259 | 74 | 258 | 15 |
| 4– | 37 | 198 | 66 | 238 | 103 | 223 | 13 |
| 5– | 60 | 196 | 122 | 235 | 182 | 221 | 9 |
| 6– | 38 | 177 | 72 | 204 | 110 | 194 | 11 |
| 7– | 32 | 149 | 66 | 217 | 98 | 192 | 12 |
| 8– | 30 | 203 | 58 | 199 | 88 | 200 | 12 |
| ≧9 | 24 | 185 | 49 | 178 | 73 | 180 | 13 |

[a]For linear trend according to storage time, $P<0.0001$.

**Table IX** Summary of the prospective biochemical epidemiological studies of beta-carotene and cancer

| *Study* | *Sex of subjects* | *Site of cancer* | *Number of: Cancer subjects* | *Number of: Controls* | *Approximate mean time to diagnosis of cancer (years)* | *Plasma (P) or serum (S)* | *Overall mean beta-carotene ($\mu g l^{-1}$) (approximate s.d.)*[e] | *Mean beta-carotene difference ($\mu g l^{-1}$). Cancer subjects minus controls (approximate s.d.)*[e] | *Published statistical significance* |
|---|---|---|---|---|---|---|---|---|---|
| Stähelin *et al.* (1984) | Male | All | 115 | 308 | 4 | P | 206 (131) | −55 (14)[d] | not given |
| Willett *et al.* (1984*a*) | Both | All | 111 | 210 | 3 | S | 1,126 (569)[a] | +34 (64) | $P=0.63$[b] |
| Nomura *et al.* (1985) | Male | 5 sites | 284 | 302 | 5 | S | 263 (252)[e] | −56 (21)[d] | $P=0.004$[f] |
| Menkes *et al.* (1986) | Both | Lung | 99 | 196 | 5 | S | 278 (234) | −40 (29) | $P=0.001$[b] |
| Present study | Male | All | 271 | 533 | 3 | S | 213 (130)[b] | −23 (9)[b] | $P=0.007$[b] |
| Overall | — | — | 880 | 1,549 | — | — | — — | −35 (7)[c] | $P=0.001$ |

[a]Total carotenids were assayed, not beta-carotene; [b] Values obtained from analysis of log beta-carotene; [c]The overall mean across studies was calculated as a mean of the individual mean differences, each weighted inversely according to its variance; [d]Mean difference not adjusted for smoking; [e]s.d. based on the 25th and 75th percentiles in controls; [f]$P$ value for linear trend in log odds ratio over quantiles of beta-carotene. ($P$ value adjusted for smoking is given as 0.04).

beta-carotene and cancer, there is an inverse association between serum beta-carotene and the risk of cancer and that the effect is present for five and more years before the diagnosis of the cancer. The study was not large enough to be able to indicate with confidence the sites of cancer for which the association applied though it is of interest that the association was strongest for lung cancer, which is consistent with the prospective dietary studies of beta-carotene intake (Bjelke, 1975; Shekelle *et al.*, 1981; Kvale *et al.*, 1983).

There are four other published prospective studies of serum beta-carotene or total carotenoids and cancer. These are summarised in Table IX together with our present results. Overall the results are consistent in showing that the average serum beta-carotene level was lower in subjects who developed cancer than in those who did not. In one study (Willett *et al.*, 1984), total carotenoids were measured rather than beta-carotene alone and showed no significant difference between cancer subjects and controls. In the reports of two of the other studies (Stähelin *et al.*, 1984 and Nomura *et al.*, 1985) the mean beta-carotene differences between cancer subjects and controls were not adjusted for smoking habit. Since smokers tend to have lower serum beta-carotene concentrations than non-smokers and smoking is also associated with the risk of cancer the mean differences between cancer subjects and controls given for these two studies is likely to over-estimate the differences in beta-carotene that relate, independently of smoking habit, to the risk of cancer. Nonetheless, the results of the two remaining studies in Table IX (Menkes *et al.*, 1986 and the present study) which allowed for smoking habits showed a similar, but less marked, effect.

The observation that the inverse association between beta-carotene and the risk of cancer persists for several years before the diagnosis of cancer, and does not appear to show a greater effect in those cancer cases for which the time between blood collection and diagnosis was short, indicates that the low beta-carotene level probably precedes the development of cancer. The fact that, in our study, there were only 59 men in whom a diagnosis of cancer was made more than five years after the time of blood collection means that we cannot completely exclude the possibility that early cancer may itself influence serum beta-carotene levels but, taken with the known long-term association between beta-carotene *consumption* and cancer, it is unlikely. The inverse association could arise either directly, because beta-carotene reduces the risk of cancer, or indirectly, because beta-carotene intake is associated with the intake of another dietary component that affects the risk of cancer. Which of the two explanations is the correct one should emerge from the results of the large-scale randomised trial of beta-carotene supplementation currently in progress among physicians in the United States of America (Hennekens, 1986). Whatever the answer the association represents a most interesting epidemiological clue to the link between diet and cancer.

We thank Dr R.M. Salkeld and Dr J.P. Vuilleumier of Hoffman–La Roche, Basle, Switzerland for performing the beta-carotene assays, and Mr P. Thompson for technical assistance. We thank the Imperial Cancer Research fund and the British United Provident Association for financial support.

### References

BJELKE, E. (1975). Dietary vitamin A in human lung cancer. *Int. J. Cancer*, **15**, 561.

BJELKE, E. (1978). Dietary factors in the epidemiology of cancer of the stomach and large bowel, In *Aktuelle Ernahrungsmedizin*, Supp. 10–17. Thieme, Stuttgart.

BRESLOW, N.E. & DAY, N.E. (1980). *Statistical Methods in Cancer Research*, Vol. 1, p. 247. The analysis of case-control studies, IARC: Lyon.

BURTON, G.W. & INGOLD, K.U. (1984). Beta-carotene: An unusual type of lipid antioxidant. *Science*, **224**, 569.

BYERS, T., VENA, J., METTLIN, C., SWANSON, M. & GRAHAM, S. (1984). Dietary vitamin A and lung cancer risk: An analysis by histologic subtypes. *Amer. J. Epidemiol.*, **120**, 769.

COOK-MOZAFFARI, P.J., AZORDEGAN, F., DAY, N.E., RESSICAUD, A., SABAT, C. & ARAMESH, B. (1979). Oesophageal cancer studies in the Caspian Littoral of Iran: results of a case-control study. *Br. J. Cancer*, **39**, 293.

FOOTE, C.S. (1979). Detection of singlet oxygen in complex systems: a critique. In *Biochemical and Clinical Aspects of Oxygen*, Caughey, W.S. (ed.), p. 603. Academic Press, New York.

GREGOR, A., LEE, P.N., ROE, F.J.C., WILSON, M.J. & MELTON, A. (1980). Comparison of dietary histories in lung cancer cases and controls with special reference to vitamin A. *Nutri. Cancer*, **2**, 93.

HENNEKENS, C.H. (1986). Micronutrients and cancer prevention. *New Engl. J. Med.*, **315**, 1288.

HINDS, M.W., KOLONEL, L.N., HANKIN, J.H. & LEE, J. (1984). Dietary vitamin A, carotene, vitamin C, and risk of lung cancer in Hawaii. *Amer. J. Epidemiol.*, **119**, 227.

HIRAYAMA, T. (1979). Diet and cancer. *Nutri. Cancer*, **1**, 67.

KOLONEL, L.N., HANKIN, J.H. & YOSHIZAWA, C.N. (1987). Vitamin A and prostate cancer in elderly men: enhancement of risk. *Cancer Res.*, **47**, 2982.

KVALE, G., BJELKE, E. & GART, J.J. (1983). Dietary habits and lung cancer risk. *Int. J. Cancer*, **31**, 397.

LONG-DE, W. & HAMMOND, E.C. (1985). Lung cancer, fruit, green salad and vitamin pills. *Chin. Med J.*, **98**, 206.

MATHEWS-ROTH, M.M. (1982). Antitumor activity of beta-carotene, canthaxanthin and phytoene. *Oncology*, **39**, 33.

MACLENNAN, R., DA COSTA, J., DAY, N.E., LAW, C.H., NG, Y.K. & SHANMUGARATNAM, K. (1977). Risk factors for lung cancer in Singapore Chinese, a population with high female incidence rates, *Int. J. Cancer*, **20**, 854.

MENKES, M.S., COMSTOCK, G.W., VUILLEUMIER, J.P., HELSING, K.J., RIDER, A.A. & BROOKMEYER, R. (1986). Serum beta-carotene, vitamins A and E, selenium, and the risk of lung cancer. *New Engl. J. Med.*, **315**, 1250.

METTLIN, C. & GRAHAM, S. (1979). Dietary risk factors in human bladder cancer. *Amer. J. Epidemiol.*, **110**, 255.

METTLIN, C., GRAHAM, S., PRIORE, R., SWANSSON, M. (1981). Diet and cancer of oesophagus. *Nutri. Cancer*, **2**, 143.

MODAN, B., CUCKLE, H. & LUBIN, F. (1981). A note on the role of dietary retinol and carotene in human gastro-intestinal cancer. *Int. J. Cancer*, **28**, 421.

NOMURA, A.M.Y., STEMMERMANN, G.N., HEILBRUN, L.K., SALKELD, R.M., VUILLEUMIER, J.P. (1985). Serum vitamin levels and the risk of cancer of specific sites in men of Japanese ancestry in Hawaii. *Cancer Res.*, **45**, 2369.

PETO, R., DOLL, R., BUCKLEY, J.D. & SPORN, M.B. (1981). Can dietary beta-carotene materially reduce human cancer rates? *Nature*, **290**, 201.

PHILLIPS, R.L. (1975). Role of life style and dietary habits in risk of cancer among Seventh Day Adventists. *Cancer Res.*, **35**, 23513.

PISANI, P., BERRINO, F., MACALUSO, M., PASTORINE, U., CROSIGNANI, P. & BALDASSERONE, A. (1986). Carrots, green vegetables and lung cancer: a case-control study. *Int. J. Epidemiol.*, **15**, 463.

RETTURA, G., STRATFORD, F., LEVENSON, S.M. & SEIFTER, E. (1982). Prophylactic and therapeutic actions of supplemental beta-carotene in mice inoculated with C3HBA adenocarcinoma cells: lack of therapeutic action of supplemented ascorbic acid. *J. Natl. Cancer Inst.*, **69**, 73.

SAMET, J.M., SHIPPER, B.J., HUMBLE, C.G. & PATHAK, D.R. (1985). Lung cancer risk and vitamin A consumption in New Mexico. *Amer. Rev. Resp. Dis.*, **131**, 198.

SHEKELLE, R.B., LEPPER, M., LIU, S., MALIZA, C., RAYNOR, W.J. & ROSSOF, A.H. (1981). Dietary vitamin A and risk of cancer in the Western Electric Study. *Lancet*, **ii**, 1185.

STAHELIN, H.B., ROSEL, F., BUESS, E. & BRUBACHER, G. (1984). Cancer, vitamins, and plasma lipids: prospective Basel study. *J. Natl Cancer Inst.*, **73**, 1463.

STEHR, P.A., GLONINGER, M.F., KULLER, L., MARSH, G.M., RADFORD, E.P. & WEINBERG, G.B. (1985). Dietary vitamin A deficiencies and stomach cancer. *Amer. J. Epidemiol.*, **121**, 65.

STOCKS, P. (1958). In: *British Empire Cancer Campaign*, 35th Annual Report 1957, Part II, Suppl., 111.

TEMPLE, N.J. & BASU, T.K. (1987). Protective effect of beta-carotene against colon tumors in mice. *J. Natl Cancer Inst.*, **78**, 1211.

TUYNS, A.J., PEQUIGNOT, G. & JENSEN, O.M. (1978). Nutrition, alcool et cancer de l'oesophage. *Bull Cancer (Paris)*, **65**, 58.

VUILLEUMIER, J.P., KELLER, H.E., GYSEL, D. & HUNZIKER, F. (1983). Clinical chemical methods for the routine assessment of the vitamin status in human populations. Part I. The fat-soluble vitamins A and E, and beta-carotene. *Int. J. Vit. Nutr. Res.*, **53**, 265.

WALD, N.J. (1982). Vitamin A and cancer in humans, In *Disease and the Environment*, Rees, A.R. & Purcell, H.J. (eds) 175–190. John Wiley, Chichester.

WALD, N., BOREHAM, J. & BAILEY, A. (1986). Serum retinol and subsequent risk of cancer. *Br. J. Cancer*, **54**, 957.

WALD, N.J., CUCKLE, H.S., BARLOW, R.D., THOMPSON, P., NANCHAHAL, K., BLOW, R.J., BROWN, I., HARLING, C.C., McCULLOCH, W.J., MORGAN, J. & REID, A.A. (1985). The effect of vitamin A supplementation on serum retinol and retinol binding protein levels. *Cancer Lett.*, **29**, 203.

WALD, N., IDLE, M., BOREHAM, J., BAILEY, A. (1980). Low serum vitamin A and subsequent risk of cancer: preliminary results of a prospective study. *Lancet*, **ii**, 813.

WALD, N.J., THOMPSON, S.G., DENSEM, J.W., BOREHAM, J. & BAILEY, A. (1987). Serum vitamin E and subsequent risk of cancer. *Br. J. Cancer*, **56**, 69.

WILLET, W.C., STAMPFER, M.J., UNDERWOOD, B.A., TAYLOR, J.O. & HENNEKENS, C.H. (1983). Vitamins A, E and Carotene: effects of supplementation on their plasma levels. *Am. J. Clin. Nutr.*, **38**, 559.

WILLETT, W.C., STAMPFER, M.J., UNDERWOOD, B.A. & 6 others (1984*a*). Vitamin A supplementation and plasma retinol levels: a randomized trial among women. *J. Natl Cancer Inst.*, **73**, 1445.

WILLETT, W.C., POLK, B.F., UNDERWOOD, B.A., STAMPFER, M.J., PRESSEL, S., ROSNER, B., TAYLOR, J.O., SCHNEIDER, K. & HAMES, C.G. (1984*b*). Relation of serum vitamins A and E and carotenoids to the risk of cancer. *New Engl. J. Med.*, **310**, 430.

WU, A.H., HENDERSON, B.E., PIKE, M.C., YU C.M. (1985). Smoking and other risk factors for lung cancer in women. *J. Natl Cancer Inst.*, **74**, 747.

ZIEGLER, R.G., MASON, T.J., STEMHAGER, A. & 6 others (1984). Dietary carotene and vitamin A and risk of lung cancer among white men in New Jersey. *J. Natl Cancer*, **73**, 1429.